# More Than Meets the Eye: Scientific Rationale behind Molecular Imaging and Therapeutic Targeting of Prostate-Specific Membrane Antigen (PSMA) in Metastatic Prostate Cancer and Beyond

**DOI:** 10.3390/cancers13092244

**Published:** 2021-05-07

**Authors:** Anniina Hyväkkä, Verneri Virtanen, Jukka Kemppainen, Tove J. Grönroos, Heikki Minn, Maria Sundvall

**Affiliations:** 1 Institute of Biomedicine, Cancer Research Unit, FICAN West Cancer Center Laboratory, University of Turku and Turku University Hospital, FI-20520 Turku, Finland; anniina.e.hyvakka@utu.fi (A.H.); vervir@utu.fi (V.V.); 2 Turku Doctoral Programme of Molecular Medicine (TuDMM), University of Turku, FI-20520 Turku, Finland; 3 Turku PET Centre, University of Turku, FI-20521 Turku, Finland; jukkem@utu.fi; 4 Department of Clinical Physiology and Nuclear Medicine, Turku University Hospital, FI-20521 Turku, Finland; 5 Docrates Cancer Center, FI-00180 Helsinki, Finland; 6 Preclinical Imaging Laboratory, Turku PET Centre, University of Turku, FI-20520 Turku, Finland; tovgro@utu.fi; 7 Department of Oncology, FICAN West Cancer Center, University of Turku and Turku University Hospital, FI-20521 Turku, Finland; heikki.minn@utu.fi

**Keywords:** castration resistant prostate cancer (CRPC), molecular imaging, positron emission tomography (PET), prostate-specific membrane antigen (PSMA), radionuclide therapy, therapeutic antibodies

## Abstract

**Simple Summary:**

Prostate-specific membrane antigen (PSMA) is a transmembrane protein that is overexpressed in prostate cancer and correlates with the aggressiveness of the disease. PSMA is a promising target for imaging and therapeutics in prostate cancer patients validated in prospective trials. However, the role of PSMA in prostate cancer progression is poorly understood. In this review, we discuss the biology and scientific rationale behind the use of PSMA and other targets in the detection and theranostics of metastatic prostate cancer.

**Abstract:**

Prostate cancer is the second most common cancer type in men globally. Although the prognosis for localized prostate cancer is good, no curative treatments are available for metastatic disease. Better diagnostic methods could help target therapies and improve the outcome. Prostate-specific membrane antigen (PSMA) is a transmembrane glycoprotein that is overexpressed on malignant prostate tumor cells and correlates with the aggressiveness of the disease. PSMA is a clinically validated target for positron emission tomography (PET) imaging-based diagnostics in prostate cancer, and during recent years several therapeutics have been developed based on PSMA expression and activity. The expression of PSMA in prostate cancer can be very heterogeneous and some metastases are negative for PSMA. Determinants that dictate clinical responses to PSMA-targeting therapeutics are not well known. Moreover, it is not clear how to manipulate PSMA expression for therapeutic purposes and develop rational treatment combinations. A deeper understanding of the biology behind the use of PSMA would help the development of theranostics with radiolabeled compounds and other PSMA-based therapeutic approaches. Along with PSMA several other targets have also been evaluated or are currently under investigation in preclinical or clinical settings in prostate cancer. Here we critically elaborate the biology and scientific rationale behind the use of PSMA and other targets in the detection and therapeutic targeting of metastatic prostate cancer.

## 1. Introduction

Prostate cancer is the second most commonly diagnosed cancer in men and the sixth leading cause of cancer-related deaths among men worldwide although incidence and mortality of prostate cancer vary depending on the country [1]. Incidence and mortality rates have been on the decline or have stabilized recently particularly in high-income countries. Although the prognosis for localized prostate cancer is good, with 5-year survival rates above 90%, recurrence can occur after radical therapy, and many patients have metastases at the time of primary diagnosis [2]. Conventional ^99m^Tc-bone scintigraphy and computed tomography (CT) showed limited diagnostic accuracy to detect and localize disease or treatment response in many cases. In particular, they are insensitive in the event of biochemical relapse with a rising prostate-specific antigen (PSA) after radical treatment, presenting an unmet need for better methods.

The first-line treatment option for metastatic prostate cancer is androgen deprivation therapy (ADT) that can be combined with docetaxel chemotherapeutic agent or drugs interfering with androgen signaling in early castration naïve state, but eventually, the lethal castration-resistant disease develops [2,3]. The therapeutic landscape of metastatic prostate cancer has evolved during recent years to extend survival. Prostate-specific membrane antigen (PSMA) is a promising clinically validated target for expression-based imaging and therapies, but a deeper understanding of the underlying biology is needed to optimize its use. Evidence from the first randomized phase III trial supports the benefit of therapeutically targeting PSMA [4]. Here we will critically discuss the scientific rationale behind the use of PSMA-directed molecular imaging and therapeutics and compare it to other potential targets evaluated in imaging of metastatic prostate cancer to guide management.

## 2. The Role of PSMA in the Biology of Prostate Cancer

### 2.1. Gene and Protein Structure of PSMA

PSMA, also known as glutamate carboxypeptidase II (GCPII), N-acetylaspartylglutamate peptidase, and N-acetyl-L-aspartyl-L-glutamate peptidase I (NAALADase I), is a type II transmembrane glycoprotein encoded by *FOLH1* (folate hydrolase 1) gene mapped to chromosome 11 short arm (11p11–11p12) and contains 19 exons, encoding a protein of 750 amino acids and a molecular weight of approximately 100 kDa [5,6].

**Figure 1:**
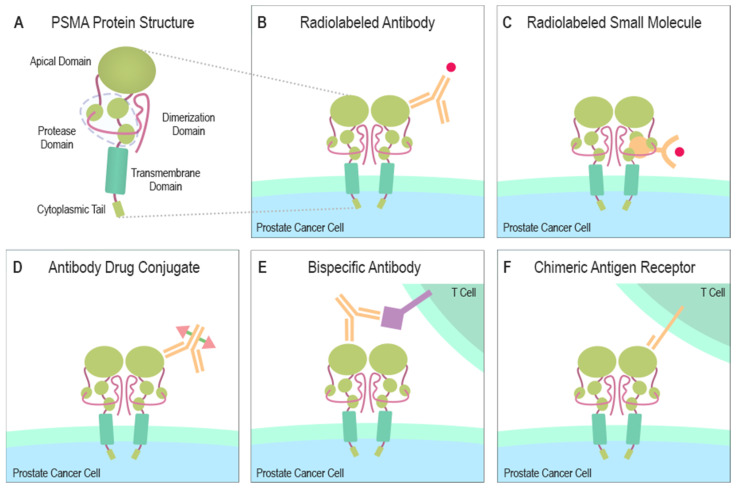
Structure of PSMA and PSMA-targeting therapeutical modalities. (**A**) The large extracellular portion of PSMA contains the protease domain that cleaves glutamate from NAAG and polyglutamated folates. The cytoplasmic tail interacts with several proteins some of which can induce the endocytosis of PSMA. (**B**) A radionuclide capable of emitting ionizing radiation due to radioactive decay can be combined with a PSMA-specific antibody to create a PSMA-targeting cytotoxic molecule. (**C**) A similar function is gained when a small molecule, naturally bound by PSMA, is linked to a radionuclide emitting ionizing radiation. (**D**) ADCs are altered antibodies carrying therapeutic agents to the targeted protein. Current ADC trials are testing PSMA-targeting antibodies carrying microtubule-disrupting agents (Table 1). (**E**) Bispecific antibodies can be designed to target PSMA and simultaneously attach to CD3 or CD28 expressed by T cells. (**F**) Autologous or allogeneic T cells can be engineered to express PSMA-targeting CARs. Current clinical trials are also studying similarly engineered NK cells. CAR T or NK cells can be designed to ignore immunosuppressive signals from the tumor microenvironment by making them insensitive to certain molecules e.g., PD-1. Abbreviations: PSMA = prostate-specific membrane antigen, NAAG = N-acetylaspartylglutamate, ADC = antibody-drug conjugate, CD3 = cluster of differentiation 3, CD28 = cluster of differentiation 28, CAR = chimeric antigen receptor, NK = natural killer cell, PD-1 = programmed cell death protein 1.

PSMA contains a catalytic domain responsible for both NAALADase and folate hydrolase activity and belongs to the M28 metalloprotease family that contains aminopeptidases and carboxypeptidases. PSMA protein has a large extracellular domain, a short transmembrane domain, and a short cytoplasmic tail [6,7,8]. The extracellular domain consists of three subdomains: the protease, the apical domain, and the dimerization domain, which are all necessary for substrate binding [9,10] (Figure 1A). In the binding cavity of PSMA, the pharmacophore pocket stabilizes glutamate-like moieties using polar and van der Waals interactions [11]. The active site of PSMA contains two catalytic zinc ions coordinated by His377, His553, Asp387, Asp453, and Glu425 [10,12]. The short intracellular domain contains an internalization motif and interacts with proteins, such as caveolin-1, clathrin, and clathrin adaptor protein 2, enabling PSMA endocytosis via caveolae-dependent mechanisms and via clathrin-coated pits [13,14,15]. Additionally, an interaction between actin-binding protein Filamin A (FLNa) and the cytoplasmic tail of PSMA has been shown to decrease the internalization and the enzymatic NAALADase activity of PSMA in vitro [16].

### 2.2. Expression and Function of PSMA in Normal Tissues

In general, only very low levels of PSMA protein expression have been detected in healthy tissues such as the kidney, intestine, salivary glands, and brain, and it seems that prostatic epithelium is the only tissue to express a significant level of PSMA [17,18]. Despite more than three decades of extensive research, the exact biological role of the human PSMA protein is not fully understood. Several independent research groups have inactivated the PSMA-encoding gene *Folh1* in mice to understand the physiological role of the mouse homolog of human PSMA [19,20,21,22]. In mice, PSMA is particularly expressed in the brain and kidney according to the Northern blot analyses [23]. Bacich et al. [19] report that PSMA null mice (intron-exon boundary sequences of exons 1 and 2 deleted and stop codons inserted in exon 1 and 2) have similar N-acetyl-L-aspartyl-L-glutamate (NAAG) levels in the brain as compared to wild-type (WT) mice, suggesting genetic redundancy. The null mice developed normally to adulthood but in comparison to WT showed lower susceptibility to peripheral neuropathies and traumatic brain injury [24]. In accordance, Gao et al. [22] reported PSMA null mice (deletion of exons 3 to 5) with normal breeding performance and no obvious phenotype. Vorlová et al. [25] produced PSMA deficient mice by inactivating (deleted exon 11) *Folh1* gene using transcription activator-like effector nuclease (TALEN)-mediated mutagenesis. They confirmed that PSMA protein was not expressed and NAAG hydrolyzing activity was lowered, but the PSMA-deficient mice bred and developed normally. They reported that PSMA-deficient aged mice might have an increased propensity for enlarged seminal vesicles compared to WT, but no other obvious phenotype in the urogenital system. In contrast, Tsai et al. [20] reported that PSMA null mice (deletion of exons 9 and 10) died during embryogenesis. The same research group later generated mice also by using the strategy reported by Bacich et al., and again reported that PSMA knockout is embryonically lethal [20,21]. They suggest that PSMA expression in embryonic stem cells might be important at very early stages of embryonic development, but the reason for the discrepancy with other studies is not clear [21].

In humans, the functional role of PSMA is context-dependent and tissue-specific. PSMA has an enzymatic activity to release glutamate from the substrate as a folate hydrolase and NAALADase [26,27,28]. In the intestine, PSMA detaches glutamates from the C-terminal end of poly-*γ*-glutamate of the dietary folic-polyglutamates enabling absorption of monoglutamated folates into the enterocytes [29,30]. In the nervous system, PSMA modulates neuronal signaling by catalyzing the hydrolysis of the neurotransmitter NAAG yielding N-acetyl-aspartate (NAA) and free L-glutamate [31]. Glutamate is the major excitatory neurotransmitter in the nervous system. The released glutamate activates postsynaptic metabotropic (mGluR) and ionotropic (iGluR) receptors of glutamate [32]. In conclusion, although PSMA is a multifunctional protein expressed in some healthy tissues, the most significant expression levels are found in humans in the prostatic epithelium.

### 2.3. Expression and Function of PSMA in Malignancies

PSMA was first characterized in 1986 by the murine monoclonal antibody 7E11, delivered from mice immunized with partially purified, cell membrane fractions isolated from the human prostate adenocarcinoma cell line LNCaP [7]. Later PSMA has been implicated a role in diseases, such as amyotrophic lateral sclerosis (ALS), schizophrenia, multiple sclerosis, inflammatory bowel disease (IBD), and cancer [33,34,35,36]. In cancer, PSMA expression has been detected on the endothelial cells of the neovasculature of several solid tumors, such as renal, bladder, gastric, and colorectal cancer as well as prostate cancer [17,37,38,39]. In prostate cancer, PSMA is highly overexpressed at the protein level in cancer cells when compared to normal prostate tissue [40]. Expression level correlates with the aggressiveness of the disease and high PSMA expression levels have been associated with hormone-refractory and metastatic prostate cancer [18,40,41,42,43,44]. However, the expression of PSMA in prostate cancer can be very heterogeneous, and some primary tumors and metastases are negative for PSMA [7,18,40,45]. The use of different antibodies in immunohistochemistry with different epitopes can also challenge the interpretation of results [38]. Heterogenous expression of PSMA may be explained by regulation of PSMA by local biological factors and tumor cell microenvironment.

Interestingly, by using a PSMA targeting antibody (YPSMA-1), an inhibitor of the enzymatic activity (2-PMPA) or PSMA null mice as models, it was shown that PSMA regulates endothelial cell invasion into the extracellular matrix without significantly affecting viability, proliferation, or morphogenesis [46]. This suggests that PSMA regulates angiogenesis depending on the enzymatic activity. Mechanistically, laminin-specific integrin β_1_ activation promotes activation of p21-activated kinase 1 (PAK) to interact with FLNa, disrupting PSMA-FLNa interaction and the enzymatic activity of PSMA. Disruption of PSMA-FLNa interaction causes a reduction in both integrin β_1_ signaling and PAK activation creating a negative feedback loop. Later, it was recognized that PSMA takes part in a pathway producing pro-angiogenic fragments from extracellular protein laminin, promoting angiogenesis by regulating integrin β_1_ signaling in endothelial cells [47]. In conclusion, high PSMA expression levels have been detected specifically in prostate cancer cells and in the neovasculature of some solid tumors, making it an attractive target for molecular imaging and therapeutics.

### 2.4. Functional Role of PSMA in Prostate Cancer

Although PSMA was originally identified in a prostate cancer LNCaP cell line and early on linked to prostate cancer aggressiveness, PSMA is not expressed in many other commonly used commercially available prostate cancer cell lines and preclinical publications have been in part contradictory, demonstrating inhibition of PSMA to either promote or prevent invasion in vitro [48,49]. First, in 2005, Ghosh et al. [48] showed that transfecting PSMA negative PC3 cells to overexpress wild-type PSMA reduced invasiveness and knocking down endogenous PSMA in LNCaP cells increased invasiveness in the Matrigel invasion assay. Later, they showed using the PSMA-overexpressing transgenic mouse model, that PSMA overexpression increased prostate cancer cell growth in prostate recombinants [49]. Moreover, they repeated the Matrigel invasion assay using low levels of folate and surprisingly observed that ectopic expression of PSMA induced invasiveness in PC3 cells, suggesting that folate levels might modulate the functional consequence to inhibition of PSMA in prostate cancer cells—at least within in vitro. Colombatti et al. also suggested that treating LNCaP cells endogenously expressing PSMA with antibodies against PSMA induced proliferative MAPK pathway activation [50]. The abundance of PSMA in prostate tissue allows increased hydrolysis of the polyglutamated folates yielding glutamate and folate monoglutamate enabling the intake of folates into the cell via proton-coupled folate transporters (PCFT), reduced folate carriers (RFC), or possibly by PSMA itself [51,52]. Folate is crucial for one-carbon metabolism and is involved in the synthesis of DNA and RNA, and amino acid metabolism [51]. Some studies suggest that decreased folate levels cause epigenetic changes, DNA breaks, translocations, and uracil misincorporation into DNA suggesting a possible carcinogenic role for low folate levels [53,54,55,56] but the role of folate in cancer seems complex and also conflicting results exist, suggesting that decreased folate levels are a protective factor against prostate carcinogenesis [57]. Based on a meta-analysis, high blood folate levels associate with increased risk of prostate cancer although the increased dietary and total folate intake do not appear significantly to associate with prostate cancer risk [53].

Folic acid is a synthetic, fully oxidized, monoglutamated version of folate—found for example in vitamin supplements—that can directly be transported into cells while polyglutamated folates have to be hydrolyzed into monoglutamates before being absorbed in the digestive tract. It has been suggested that polyglutamated folate in turn could become available as a substrate for PSMA in particular if intracellular molecules were released by cancer cells undergoing necrotic cell death [58]. Interestingly, tumor necrosis in radical prostatectomies was associated with aggressive features [59]. Treatment of LNCaP cells endogenously expressing PSMA with folic acid induced activation of PI3Kβ-Akt pathway, which was not observed in the presence of inhibitors of either PSMA, PI3Kβ, or mGluRI activity [60]. This suggests that PSMA is capable of activating the PI3K-Akt cell survival pathway in prostate cancer and surprisingly finds mGluRI as the major mediator of folate-induced PI3Kβ-Akt pathway activation by PSMA. Glutamate is a necessary metabolic precursor for other amino acids and nucleotides and is involved in a variety of different transporter and receptor systems that activate proliferative signaling pathways. Glutamate also mobilizes calcium from the endoplasmic reticulum via the activation of mGluR [60]. Expression of glutamate transporters and many of the different iGluRs and mGluRs have been reported in LNCaP and PC3 prostate cancer cell lines [61]. Glutamate deprivation or blockade with mGluR1-antagonist results in significantly decreased cell proliferation, migration, and invasion of prostate cancer cells, leading to apoptotic cell death, demonstrating a potentially important role of glutamate pathway for prostate cancer growth [62]. In prostate cancer patients, serum glutamate levels directly correlate with Gleason Score and aggressiveness [62]. Moreover, high mGluR1 levels in primary and metastatic prostate cancer tissue when compared to benign prostate tissue samples have been detected by immunohistochemistry [62].

Caromile et al. [63] crossed PSMA null mice with a TRAMP transgenic mouse model to investigate in vivo effects of PSMA in prostate carcinogenesis. Prostate cancer progression in a TRAMP transgenic mouse model was less aggressive in PSMA deficient background, suggesting a direct role for PSMA in prostate carcinogenesis. PSMA positive tumors were bigger and had a higher microvessel density when compared to tumors in the PSMA knockout animals. Mechanistically, PSMA was shown to interact intracellularly with a scaffolding protein receptor for activated C kinase 1 (RACK1) and disrupt the interaction between insulin-like growth factor 1 receptor (IGF1-R) and integrin β_1_, resulting in a switchlike change from activation of the proliferation-associated MAPK pathway to activation of a cell survival-associated PI3K-AKT pathway. This suggested one mechanism of how PSMA overexpression could drive prostate cancer growth and indicated that disrupting the interaction between PSMA and the scaffold might have therapeutic potential for patients with PSMA-positive prostate cancer. Mutations targeting phosphatase and tensin homolog (PTEN) or other components of the PI3K-AKT pathway are often found in prostate cancer and crosstalk between the PI3K-AKT pathway and AR signaling has been described [64,65]. This reciprocal feedback regulation may be involved in resistance to therapeutics resulting in AR or PI3K-AKT inhibition, resulting in the activation of the other pathway when the other one is inhibited [64,65]. The integrin β_1_-mediated mechanism linking PSMA to the PI3K-AKT pathway may thus result in a similar reciprocal feedback regulation towards AR signaling. Interestingly, RACK1 has also been shown to interact with AR and inhibit its function, putatively also connecting PSMA to AR signaling [66].

**Figure 2:**
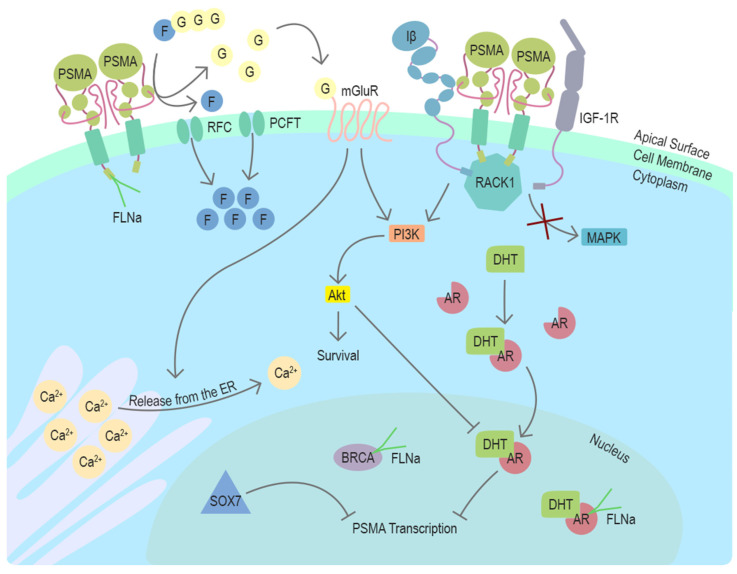
Function and regulators of PSMA in prostate cancer cells. PSMA is a transmembrane protein expressed on the surface of prostate cancer cells. In the dimeric form, PSMA can enzymatically hydrolyze glutamated folates producing glutamate and folates that can enter the cell through RFC or PCFT. Activation of mGluR by glutamate induces the release of calcium from the ER and activates cell survival associated with the PI3K-Akt pathway. In the absence of PSMA, Integrin β (Iβ) and IGF1-1R interact intracellularly with a scaffold protein RACK1 leading to the activation of the MAPK pathway. This interaction is disrupted by PSMA, changing the pathway activation from MAPK to PI3K-Akt in a switchlike manner. DHT is the physiological activator of AR. PSMA transcription is suppressed by AR after activation by DHT. Another potential repressor of PSMA transcription in prostate cancer cells is SOX7, which has been shown to bind to PSME. Cytoskeleton-related protein FLNa interacts with the intracellular domain of PSMA. FLNa interestingly also interacts with AR and BRCA1, and BRCA2. Abbreviations: PSMA = prostate-specific membrane antigen, FLNa = filamin A, G = glutamate, F = folate, RFC = reduced folate carrier, PCFT = proton-coupled folate transporter, mGluR = metabotropic glutamate receptor, ER = endoplasmic reticulum, Iβ = integrin β, IGF-1R = insulin-like growth factor 1 receptor, RACK1 = receptor of activated protein C kinase 1, MAPK = mitogen-activated protein kinase, PI3K = phosphoinositide 3-kinase, Akt/PKB = protein kinase B, DHT = dihydrotestosterone, AR = androgen receptor, PSME = PSMA enhancer, BRCA = breast cancer type 1 or 2 susceptibility protein, SOX7 = SRY-Box transcription factor 7, Ca^2+^ = calcium ion.

In conclusion, recent studies using different model systems including transgenic mouse models with PSMA null background have elucidated several distinct mechanisms associating PSMA with survival-related signaling pathways with connections to the androgen receptor (AR) signaling in prostate cancer (Figure 2). The newly discovered mechanisms provide new context also for the earlier studies and cooperatively suggest a role for PSMA as an important participant in multiple stages of prostate cancer progression. 

### 2.5. Regulation of PSMA Expression and Activity in Prostate Cancer

PSMA expression is highly regulated in a prostate cell and prostate cancer cell-specific manner [67]. A prostate tissue-specific PSMA enhancer (PSME) is involved in this process [68], but surprisingly, little is known about transcriptional regulators in detail in prostate tissue. PSMA expression is regulated by the NFATc1 transcription factor which binds to PSME, activating the transcription of PSMA [69]. A SOX7 transcription factor is another transcription factor shown to bind to PSME and in turn, negatively regulate PSMA expression. However, the role of NFAT1c or SOX7 in regulating PSMA expression in the prostate cancer context is not characterized. Interestingly, downregulation of SOX7 in prostate cancer has been found in castration-resistant prostate cancer (CRPC) [70].

Androgen signaling and subsequent AR activation is a hallmark of prostate cancer progression and surprisingly has long been known to downregulate PSMA [18,71,72]. Androgens have been suggested to suppress the transcription of the PSMA gene acting via both promoter and enhancer regions [71]. Interestingly, a fragment of FLNa can translocate to the nucleus and repress the transactivator function of AR [73], suggesting that FLNa might also be involved in the regulation of PSMA expression. FLNa has been found more in the cytoplasm than in the nucleus in metastatic prostate cancer when compared to localized prostate cancer, and cytoplasmic FLNa is linked to invasion [74]. Murga et al. [75] supported the previous result by showing that castration in combination with AR signaling inhibitors, such as enzalutamide upregulated PSMA expression in androgen-dependent LNCaP cells. They also demonstrated that PSMA-targeting antibody-drug conjugate was synergistic with AR signaling inhibitors. Hope et al. [76] reported first in human experience that AR inhibition increases PSMA expression in prostate cancer metastases and increases the number of lesions visualized on PSMA-based positron emission tomography (PET) imaging. After that, many studies have reported the flare phenomenon increasing the expression of PSMA after ADT [77]. The majority of studies suggest that short-term ADT induces temporary up-regulation of PSMA expression but under prolonged androgen deprivation, down-regulation of PSMA expression is detected and finally the androgen-resistant tumors have overexpression of PSMA [78,79,80]. It is thought that the expression of PSMA may depend on the patient’s castration status. It is possible that the “MAPK/PI3K-Akt switch” function of PSMA could help the prostate cancer cells to try to survive castration when AR activation is suppressed.

Overexpression of PSMA has been associated with DNA repair defects in tumors [81]. However, the mechanisms behind the overexpression of PSMA in this context are likely multifactorial involving several cellular processes and are not yet well understood. PSMA-interactor FLNa interestingly also interacts with BRCA1 and BRCA2 which are among the most frequently found DNA damage repair (DDR) genes mutated in metastatic prostate cancer [82,83], suggesting one putative link between PSMA and DDR. Taken together, AR signaling is the best known clinically validated regulator of PSMA expression, but relatively little is known of the regulation of PSMA expression on a molecular level.

## 3. PSMA as a Target for Imaging and Therapeutics in Prostate Cancer Patients

### 3.1. PSMA Based Imaging in Prostate Cancer

**Figure 3:**
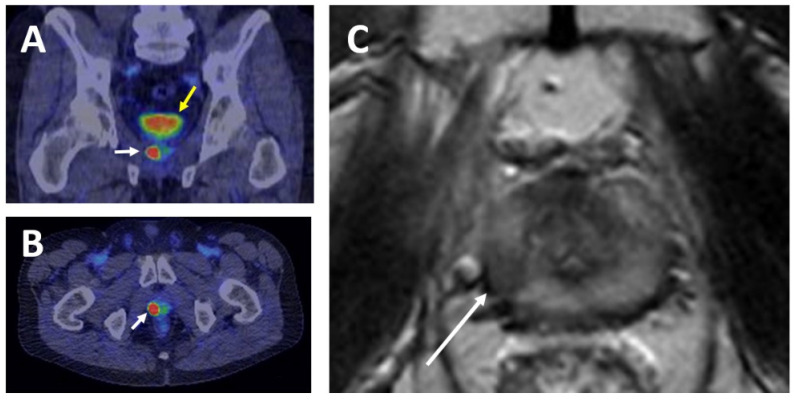
Example showing the potential of PSMA PET-CT imaging in detecting local recurrence of prostate cancer. Coronal (**A**) and axial (**B**) views of ^68^Ga-PSMA PET-CT demonstrate a recurrent right peripheral Gleason 5 + 5 carcinoma in a previously irradiated prostate (white arrow). The bladder (yellow arrow) is well visible. T2-weighted magnetic resonance imaging (**C**) indicates the presence of a PI-RADS (Prostate Imaging Reporting and Data System) grade 5 lesion or clinically significant cancer (white arrow) in the same area. Image: H. Minn, Turku PET Centre.

Conventional ^99m^Tc bone scintigraphy and CT are insensitive to detect in particular early metastases and there has been an unmet medical need for improved sensitive and specific imaging methods to guide treatment. Since the 1990s several PSMA based imaging methods have been developed. Currently, to detect PSMA, ^68^Ga-PSMA-11 is the most widely studied PET imaging ligand [84]. Several studies have been conducted in prostate cancer patients with PSMA-detecting tracers for smaller patient cohorts and meta-analyses of those studies support the potential for this imaging method in prostate cancer [84,85]. In particular, PSMA-PET outperforms ^99m^Tc bone scan in detecting bone metastases [86]. Longed evidence-based data on carefully conducted large prospective trials are now emerging. A prospective multicenter single-arm trial including 635 prostate cancer patients demonstrated the accuracy of PSMA-PET in detecting prostate cancer lesions after recurrence after previous radical surgery or radiotherapy [87]. Histopathological analysis of lesions in some patients was included to confirm the specificity of PSMA-PET to detect prostate cancer. Figure 3A–C show local organ confined PSMA-positive recurrence after definitive radiotherapy. In 2020, a prospective multicenter randomized proPSMA trial including 302 patients demonstrated that PSMA PET-CT has significantly higher accuracy when compared with conventional imaging in patients with prostate cancer with high-risk features before surgery or radiotherapy [86]. In a prospective 79 patient study, ^18^F-PSMA-1007 PET-CT despite showing non-metastatic bone lesions, similarly showed superior sensitivity when compared to conventional imaging with 14% of patients having metastases only visible by PSMA PET-CT [88]. A small cohort study tested the hypothesis of ADT enhancing the performance of PSMA PET-imaging [89]. Nine patients were imaged using ^68^Ga-PSMA-11 PET-MRI once before and three times after the administration of degarelix or firmagon. A heterogenous increase in the ^68^Ga-PSMA-11 uptake with a mean standardized uptake value (SUVmax) increase of 77% (range 8–238%) more evident in the bone metastases was observed 3–4 weeks post-ADT. The impact on performance was considered minor. One patient had more bone metastases visible due to the flare phenomenon without changing his management and ADT was determined not to interfere with imaging as no lesions disappeared within a month after initiation of ADT [89]. In parallel with PET, ^99m^Tc-based SPECT (single-photon emission computed tomography)-compatible PSMA ligands have been developed and are currently under clinical assessment [90]. ^99m^Tc-PSMA-SPECT-CT might have the potential to detect more aggressive prostate cancers, especially if PSMA PET-CT is not available.

### 3.2. PSMA Targeted Therapy Using Radiolabeled Small-Molecule Ligands or Antibodies in Prostate Cancer

PSMA can be targeted by small-molecule ligands and antibodies (Ab) labeled with radionuclides [91] (Figure 1B,C). The most promising classes of PSMA inhibitors are urea-based low molecular weight ligands [91]. Czerwińska et al. [92] have extensively reviewed different radioligands evaluated in prostate cancer. Urea-based agents, such as PSMA-617 and PSMA-I&T (I&T indicating imaging and therapy) radiolabeled with lutetium-177 (^177^Lu) have been evaluated as therapy ligands for metastatic prostate cancer patients. ^177^Lu is a β^-^-emitting radionuclide (half-life 6.7 days), which by emitting a beta particle (maximal tissue penetration 2 mm, maximum energy 0.5 MeV), induces cell death by breaking double-strand DNA [93,94]. The major dose-limiting organs for radiolabeled PSMA ligands seem to be salivary glands and kidneys [95,96]. The high uptake of PSMA in salivary glands does not seem to correspond to a high level of protein expression [97]. In salivary glands, the mechanism of uptake of PSMA is poorly understood. A recent study showed focally limited expression of PSMA to intercalated ducts in submandibular glands and concluded that high tracer accumulation must be mediated by a PSMA independent mechanism [97]. Although astrocytes express PSMA in the human brain [98], PSMA targeted imaging does not show prominent brain uptake in a normal brain because radiolabeled PSMA ligands do not cross an intact blood-brain barrier. Increased permeability of the blood-brain barrier, e.g., due to benign pathologies or less frequent brain metastases of prostate cancer, can lead to higher uptake of PSMA-radiotracer [99].

**Figure 4:**
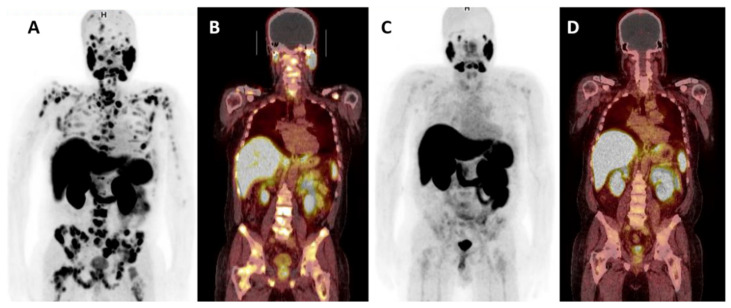
^177^Lu-PSMA-617 treatment results in metastatic castration-resistant prostate cancer. ^68^Ga-PSMA PET maximum intensity projection (MIP) images, (**A**,**C**) and coronal PET-CT (**B**,**D**) whole-body images before (**A**,**B**) and after (**C**,**D**) four cycles of ^177^Lu-PSMA-617 show almost complete disappearance of PSMA-positive metastatic tumors after radionuclide treatment in a patient with metastatic castration-resistant prostate cancer (mCRPC). Image: J. Kemppainen, Docrates Cancer Center.

^177^Lu-PSMA-617 is a small molecule that binds with high affinity to the enzymatic site of PSMA enabling highly targeted delivery of β^—^radiation. ^177^Lu-PSMA-617 therapies seem promising to men with metastatic castration-resistant prostate cancer (mCRPC) (Figure 4A–D). A comprehensive meta-analysis of 16 studies, including a total of 671 mCRPC patients demonstrated the safety and efficacy of ^177^Lu-PSMA-617 [100]. The results showed that almost half (46%) of patients had over 50% reduction of PSA and the toxicity profile was acceptable. PSMA targeting radioligands are investigated in several ongoing trials (Table 1). Results of the randomized multicenter phase II trial TheraP show more PSA responses with ^177^Lu-PSMA-617 therapy when compared to cabazitaxel treatment in mCRPC [101]. Fewer grade 3 and 4 toxicities were observed with ^177^Lu-PSMA-617 therapy when compared to cabazitaxel treatment. Importantly, the first randomized phase III trial VISION now demonstrates both overall survival benefit and better radiographic progression-free survival with ^177^Lu-PSMA-617 treatment when compared to the best standard of care in mCRPC [4]. Many large, randomized phase III trials are currently ongoing, both in hormone naïve and mCRPC to define the role of this therapy in prostate cancer (Table 1). Given the crosstalk between PSMA and AR signaling pathways, an interesting phase III PSMAddition trial is evaluating the combination of AR signaling block (castration plus standard of care androgen receptor directed therapy) and ^177^Lu-PSMA-617 (6 cycles) as a first-line therapy for hormonal naïve metastatic prostate cancer (Table 1, NCT04720157). Another interesting setting is an ongoing randomized multicenter phase II trial investigating the efficacy of two cycles of ^177^Lu-PSMA-617 upfront after the initiation of castration, followed by docetaxel chemotherapy in hormone naïve metastatic prostate cancer patients (Table 1, NCT04343885).

Actinium-225 (^225^Ac), thorium-227 (^227^Th), and copper-64 (^64^Cu) are currently evaluated in phase I trials as alternative isotopes for radiotherapy. As opposed to ^177^Lu, ^225^Ac, and ^227^Th decay alpha particles with a shorter emission path of only a few cells and high local cell kill caused by higher energy compared to β-emitting ^177^Lu and ^64^Cu [94]. Seventeen chemotherapy-naïve mCRPC patients were treated with ^225^Ac-PSMA-617, resulting in a ≥90% decline in serum PSA in 82% of patients, including 41% of patients with undetectable serum PSA who remained in remission 12 months after therapy [102]. In a pilot study tandem therapy with low-activity ^225^Ac-PSMA-617 and full-activity ^177^Lu-PSMA-617 was safe, generally well-tolerated, and showed efficacy in late-stage mCRPC patients after insufficient response to ^177^Lu-PSMA-617 monotherapy [103]. Additionally, several small-molecule ligand and antibody alternatives to PSMA-617 and PSMA-I&T are being tested (Table 1). Moreover, other promising ligands and radionuclides that show potential to attain greater efficacy are being presented or already have entered clinical trials [104,105,106]. A randomized phase II open-label study (Table 1, NCT03939689) is investigating a radioconjugate ^131^I-1095, for delivering iodine cytotoxicity selectively to the PSMA-expressing prostate cancer cells, in combination with enzalutamide in patients with mCRPC. Eligible criteria allow the inclusion of chemotherapy-naïve patients progressing under abiraterone. Another interesting setting is evaluating the efficacy of ^177^Lu-PSMA-I&T as a first-line treatment for oligometastatic prostate cancer relapse after prior surgery or external radical radiotherapy for local disease (Table 1, NCT04443062). 

Radiolabeled antibodies targeting PSMA, such as ^177^Lu-J591 have shown promising activity in a phase II clinical trial [107]. Treatment with another antibody-based radioligand, ^227^Thorium-PSMA-TTC is currently in phase I (Table 1, NCT03724747). Ludotadipep (^177^Lu-FC705) is currently in phase I and contains a PSMA binding motif as well as an albumin-binding site that is thought to allow higher concentrations in the target tissue with reduced systemic side effects (Table 1, NCT04509557). 

### 3.3. Other PSMA Targeting Therapeutic Strategies under Evaluation in Prostate Cancer

Several alternative approaches to treat prostate cancer by targeting PSMA have been tested in preclinical models and early phase clinical trials. Docetaxel nanoparticles targeting PSMA have been evaluated in a phase II study in chemotherapy naïve mCRPC patients, and both the PSA responses and the measurable disease responses were observed [108]. A prodrug, G202, consisting of a PSMA-specific peptide coupled to an analog of the potent sarcoplasmic/endoplasmic reticulum calcium adenosine triphosphatase (SERCA) pump inhibitor thapsigargin was promising in mouse xenograft models [109], but a thapsigargin-based PSMA-activated prodrug evaluated in a phase I study showed only a modest activity [110]. An antibody-drug conjugate (ADC) consisting of a humanized anti-PSMA monoclonal antibody conjugated to a toxin disrupting microtubules has been evaluated in a phase II study after progression with abiraterone or enzalutamide [111] (Figure 1D). In patients, a decline of circulating tumor cells (CTCs; ≥50%) was seen in 78% of patients, and a decline of PSA (≥50%) in 14%. Both CTC and PSA responses were more common in patients with high PSMA measured from CTCs. Overall, 51.3% of the subjects reported at least one serious adverse effect, the most common ones being dehydration, hyponatremia, and febrile neutropenia [111].

Bispecific antibodies recognize tumor antigen and help T cell recognition by also binding to CD3 on T cells (Figure 1E). Costimulatory CD28-bispecific antibodies can further enhance the antitumor activity of CD3-bispecific antibodies [112]. In the first-in-human study of a bispecific T-cell engager, PSMA and CD3 targeting pasotuxizumab (AMG 212) generated dose-dependent PSA responses in the cohort receiving intravenous administration [113]. The maximum tolerated dose was not determined because a sponsor change stopped the intravenous cohort. Interestingly, 1 of the 2 long-term responders in the 16 subject cohort had received earlier ^177^Lu radioligand therapy with no response. Several ongoing trials are now studying the role of bispecific antibodies that recognize PSMA and CD3 or CD28 (Table 1).

Engineered immune cells have been developed to treat prostate cancer [114] (Figure 1F). Early phase I trials have been reported as ongoing and are evaluating the safety and the potential of chimeric antigen receptor (CAR) T cells [115] (Table 1). In a phase I dose-escalation study, five mCRPC patients received chemotherapy conditioning and were treated with PSMA-targeted CAR T cells with a continuous infusion of low-dose interleukin 2 (IL-2) [116]. Two of the patients exhibited partial response along with 50% or 70% of PSA reduction and a third patient had a minor response. Plasma IL-2 levels were depleted by engrafted T cells, and this was suggested to restrain responses. Anti-PSMA or anti-CAR toxicities were not observed [116]. In a phase I dose-escalation study (Table 1, NCT04053062), the safety and efficacy of PSMA-CAR T cells are evaluated in 12 mCRPC patients. Another single-arm phase I study (Table 1, NCT03089203) is evaluating the safety and feasibility of dual PSMA-specific/TGF-resistant CAR-modified autologous T cells (CART-PSMA-TGFβRDN cells) without (cohort 1 and cohort 2) and with (cohort 3) intravenous cyclophosphamide in 18 mCRPC patients. Cohorts 1 and 2 allow the maximum tolerated dose of CART-PSMA-TGFβRDN cells to be identified, while cyclophosphamide in cohort 3 before CAR T cells is used as conditioning chemotherapy. New approaches to design CAR T cells might increase possibilities to develop efficient PSMA-based T cell therapies [117].

TARP is a nuclear protein expressed in prostate and breast cancer cells [118]. In a pilot study, 29 subjects were assigned to receive three different doses of a PSMA and TARP peptide vaccine with poly IC-LC adjuvant [119]. No adverse effects of grade 3 or higher were observed and as a secondary objective, PSA doubling did not occur during the mean 458-day follow-up for 10 out of 29 subjects [119]. Including more than one target antigen in one cancer vaccine may be a valid approach due to the heterogeneity of prostate cancer. In conclusion, several PSMA targeted approaches in addition to radioligands to treat prostate cancer have been tested preclinically and shown promise in early phase clinical trials, but randomized clinical data supporting efficacy is still pending. In future, one potential approach to better treatment outcomes could be to combine PSMA ligand therapy with other therapies that do not have co-toxicity.

### 3.4. Determinants of Sensitivity and Resistance to PSMA Targeting

Finding predictive factors for the sensitivity and resistance for PSMA-directed targeting is important. Patients treated with ^177^Lu-PSMA-617 with low PSMA expression by imaging have shorter survival compared to ones without PSMA-low metastases, suggesting that PSMA expression may predict response and be associate with outcomes in patients receiving ^177^Lu-PSMA-617 therapy [120]. However, many patients with PSMA-positive prostate cancer do not respond to ^177^Lu-PSMA-617. Recently, several studies have demonstrated that some patients with prostate cancer have germline and/or somatic mutations in DDR genes in their tumors [121,122,123], most commonly targeting the *BRCA2* gene, which associates with poor prognosis and more aggressive disease [124,125]. Other most frequently mutated DDR genes in prostate cancer are *CHEK2*, *ATM,* and *BRCA1* [121,122,123]. The prevalence of germline DDR mutations in primary prostate cancer is approximately 11% [121], whereas in mCRPC above 20% of patients have either germline or somatic DDR mutations in their tumors [122,123]. DDR is an essential pathway for the survival of both malignant and normal prostate cells after DNA damage. Paschalis et al. [81] reported that deleterious DDR aberrations are associated with the increased expression levels of PSMA in mCRPC patients. This suggests that the biological consequences of deleterious aberrations in DDR genes may promote, as one factor, the overexpression of PSMA, and putatively regulate sensitivity to PSMA-targeting therapeutics. Mechanisms behind the putative crosstalk between DDR pathways and PSMA are not well understood and warrants further mechanistic studies. Preclinical in vivo data suggests some candidate mediators of resistance to PSMA-targeting radioligands [126], but the role of these and other putative factors in patients should be investigated. Moreover, prospective studies evaluating the association between genomic changes in DDR genes and putative other factors in the connection with PSMA expression as well as in the connection with sensitivity to PSMA-targeting theranostics are needed.

## 4. Other Radioligands and Targets in Prostate Cancer in Comparison with PSMA Based Targeting

### 4.1. Radioligands Detecting Dependencies on Metabolic Pathways

Altered glucose metabolism, including increased uptake of glucose and a preference for aerobic glycolysis instead of oxidative phosphorylation even in the presence of oxygen, is considered one of the most extensively characterized metabolic hallmarks of many cancers [127]. However, ^18^F-fluorodeoxyglucose (^18^F-FDG) shows typically only a low uptake in prostate cancer due to slow tumor growth especially in the early stages of the disease [128]. Interestingly, PSMA-low prostate tumors with neuroendocrine features have been suggested to express high levels of glucose transporters and hexokinases, and due to increased uptake of glucose, ^18^F-FDG-PET could be an efficient imaging method for these prostate cancers [129,130,131,132]. The same phenomenon is seen in neuroendocrine tumors of gastrointestinal origin when they show aggressive growth patterns and thus they warrant more studies to evaluate the potential of ^18^F-FDG for detecting some subtypes of prostate cancer. ^18^F-FDG might also have a role together with PSMA-imaging in selecting patients for PSMA targeted therapy. MCRPC patients with discordant imaging findings in metastases with high ^18^F-FDG uptake with low or non-existent PSMA-uptake are not considered candidates for molecular radiotherapy [133].

Radiolabeled choline was until recently the most studied PET-CT tracers before PSMA became in Europe the predominant choice for molecular imaging of prostate cancer. Choline PET-CT imaging demonstrated its clinical value by showing a high accuracy for restaging patients with biochemical relapse after radical prostatectomy. Choline PET-CT imaging is based on detecting increased lipid biosynthesis and expression of choline in prostate cancer cells [134]. Choline is an essential nutrient for all cells as well as prostate cancer cells because choline has a function in the biosynthesis of the membrane phospholipids. In a meta-analysis of 609 patients, ^18^F-choline PET-CT showed a pooled sensitivity of 0.62, and specificity of 0.92 for detecting pelvic lymph node metastases, and more patients had positive findings in comparison to bone scintigraphy [135]. The specificity for primary staging was not optimal due to high tracer uptake in prostate hyperplasia [136]. However, ^68^Ga-PSMA-PET-CT has overall better detection rates in prostate cancer patients compared to ^18^F-choline or ^11^C-choline PET-CT [137,138,139,140].

^11^C-acetate is a PET tracer that exploits the upregulation of fatty acid synthase in prostate cancer. Acetate is a naturally occurring substrate for fatty acid synthesis in acetyl-CoA form. Watt et al. [141] have showed increased fatty acid uptake and significant lipidomic remodeling in human prostate cancer. Their results confirmed previously showed findings that changes in fatty acid uptake are explained, at least in part, by upregulation of the fatty acid transporter CD36, which could be a potential target in prostate cancer. Lipid metabolism has also been suggested to play a crucial role in the metastasis process [142]. However, in prostate cancer patients the diagnostic performance was better for ^68^Ga-PSMA-11 PET than ^11^C-acetate PET in detecting metastatic lesions [143].

Recent research suggests that alterations in and increased dependency on glutamine metabolisms might be an important hallmark of certain cancers [127]. Glutamine tracers ^11^C-Glutamine and ^18^F-fluoroglutamine have shown potential for human use in cerebral gliomas [144]. Cohen et al. [145] demonstrated PET imaging of glutamine metabolism in a clinical trial of metastatic colorectal cancer using ^11^C-Glutamine and ^18^F-FSPG (^18^F-(S)-4-(3-fluoropropyl)-L-glutamic acid) in four patients without toxicity or observed adverse events. Park et al. [146] published the clinical evaluation of ^18^F-FSPG for PET imaging in 10 patients with newly diagnosed and 10 patients with recurrent prostate cancer demonstrating that ^18^F-FSPG is a promising tumor imaging agent for PET with high a detection rate and a favorable biodistribution. It has been suggested that targeting the glutamine metabolism might also be a potential treatment for numerous types of cancers, including prostate cancer [147], but more understanding of glutamine metabolism in the context of prostate cancer is needed. Amino acid metabolism and transport systems, such as ASCT2 or LAT transporter, are also upregulated in prostate cancer [148,149,150]. Both synthetic and natural radiolabeled amino acids have been utilized for prostate cancer imaging. One of the most extensively investigated for prostate cancer is FACBC (Fluciclovine; anti-1-amino-3-^18^F-fluorocyclobutane-1-carboxylic acid) which is a synthetic analog of the amino acid L-leucine transported into cells via amino acid transporters [150,151]. However, it seems that PSMA-PET is superior compared to ^18^F-fluciclovine-PET for detecting biochemical recurrence in prostate cancer [152].

^18^F-NaF PET/CT has shown promise in detecting bone metastases and has been suggested to, e.g., have a role in the follow-up of therapy responses in bone predominant metastatic disease, but the obvious disadvantage is that its diagnostic power is limited to bone metastases only [153]. Uptake in non-cancer lesions in bone and lack of added clinical value compared to bone scintigraphy may limit the use of ^18^F-NaF PET/CT in clinical practice [154]. Some discordance of metastatic bone lesions have been noted between PSMA and NaF-imaging and might be related to the progression of the disease from castration sensitive to resistant forms of the disease [155,156]. Currently, it is not known whether metastatic prostate cancer patients have significant clinical benefit from NaF-imaging e.g., in case of oligometastatic disease or in cases of false-positive rib findings commonly occurring in PSMA imaging. Zhou et al. [157] showed in a meta-analysis that PSMA PET/CT performed insignificantly better in per-patient sensitivity and specificity in the detection of bone metastases compared to NaF PET/CT but NaF was superior to PSMA-PET/CT in per-lesion specificity. In conclusion, PSMA-PET seems superior compared to many metabolic radioligands tested in prostate cancer although the potential of some ligands such as ^18^F-FDG-PET should be evaluated in some subtypes of prostate cancers and PSMA-low prostate tumors.

### 4.2. Other Radioligands and Theranostics Targeting Cell Surface Molecules or Receptor Functions in Tumor Microenvironment or in Cancer Cells

Bombesin receptors (gastrin-releasing peptide receptors, GRPRs) are G-protein coupled receptors that are highly overexpressed in several human tumors, such as prostate cancer, lung cancer (small-cell and non-small-cell), breast cancer, and renal cancer [158], in line with PSMA are amenable for theranostic use [159]. Bombesin increases the growth of human prostate cancer cells and activates AR [160,161]. Kähkönen et al. [162] have demonstrated that ^68^Ga-labeled bombesin (^68^Ga-RM2; BAY86-7548) had high cancer-binding specificity and significantly higher uptake in prostate cancer than benign tissue. ^68^Ga-RM2 was also well-tolerated. ^68^Ga-RM2 was also promising in detecting biochemical recurrence [163]. However, high interindividual variability of agreement between the uptake of this radioligand and histopathology has been observed [164]. In a study comparing ^68^Ga-PSMA-11 with ^68^Ga-RM2, no PSMA-negative patients showed RM2-accumulation [165]. Both ^68^Ga-PSMA-11 and ^68^Ga-RM2 identified the same cases of local recurrence, but ^68^Ga-RM2 scans were negative in some of the patients with PSMA-positive bone or lymph node metastases. ^68^Ga-RM2 scans did reveal some additional metastatic bone lesions but did not affect the line of treatment [165]. Theranostic ^177^Lu-RM2 therapy was well tolerated in mCRPC patients with the pancreas being the critical organ [166]. Interestingly, heterodimeric ^177^Lu-radiopharmaceuticals simultaneously targeting PSMA and Bombesin (Lu-DOTA-iPSMA-Lys-BN) have been synthesized and shown to be taken up by human PC3 and LNCaP prostate cancer cells [167]. Dual targeting might be valuable due to the heterogenic nature of metastatic cancers.

Cancer-associated fibroblasts (CAFs) are important non-mutated cellular components of the tumor microenvironment modulating angiogenesis, cancer metastasis, and therapy responses [168]. CAFs express fibroblast activation protein (FAP) that can be targeted with ^68^Ga-radiolabeled quinoline-based inhibitors of FAP (FAPI) [169,170]. ^68^Ga-FAPI has shown promise in detecting metastasis of various cancer types, including PSMA-negative prostate cancer metastases [169,170]. In conclusion, the potential of ^68^Ga-RM2 and ^68^Ga-FAPI in detecting PSMA-negative prostate cancer warrants further research.

## 5. Conclusions and Future Perspectives

Improved molecular imaging methods for prostate cancer have now emerged to guide treatment. First larger prospective clinical trials have been reported and support the use of PSMA-based PET imaging to detect prostate cancer metastases, resulting in FDA approval of ^68^Ga-PSMA-11 for this indication at the end of 2020. Future challenges with imaging approaches to optimize molecular therapies include uneven distribution and implementation of technologies, lack of proper validation, and inconsistent terminology in quantitative imaging biomarkers [171]. Tumor-specific (epi)genetic cues likely modify the dependencies of cancer cells on particular metabolic and other pathways, suggesting that optimal use of imaging ligands can depend on individual factors of the tumor. Thus, future studies should include the analyses of (epi)genetic and proteomic information of individual tumors when evaluating imaging ligands.

Results of a recently reported randomized phase III trial of ^177^Lu-PSMA-617 compared to the standard of care prove the efficacy of PSMA-targeting in metastatic prostate cancer and highlight the potential of PSMA as a target for molecular radiotherapy [4]. A variety of different therapeutic approaches based on PSMA have been tested. However, they all more or less rely on the same rationale that PSMA specifically tags malignant prostate cells, and this can be exploited by guiding anti-tumor agents or cells to the PSMA expressing cancer cells. The correlation between PSMA expression and tumor grade as well as preclinical evidence hints that PSMA has tumorigenic functions, but it is not known how much the inhibition of PSMA function accounts for the effects observed with PSMA-targeted therapeutics, including radioligands and toxins, in patients. Nevertheless, mechanisms described earlier reveal several other potential therapeutic targets in prostate cancer, including inhibition of enzymatic activity or downstream targets of mGluRs, or PI3K-Akt signaling. AR signaling and PSMA-connected PI3K-AKT signaling may also upregulate each other when inhibited [64]. Combining androgen blockade with simultaneous targeting of PSMA-associated Akt activation should intercept one path to survival and castration resistance. Currently known cytoplasmic protein interactions of PSMA have also interesting interconnecting mechanisms that may present future targets and search for other putative novel interactors and molecular mechanisms regulated by PSMA in prostate cancer context is warranted. Co-operating factors and targetable vulnerabilities in PSMA-positive cell models could be searched after with CRISPR-Cas9 knockout screens, for example.

DDR aberrations are associated with higher PSMA expression levels in prostate cancer [81]. Poly (ADP-ribose) polymerase (PARP) inhibitors target the cancer cells with DDR mutations and subsequent accumulation of double-stranded breaks, and simultaneous radioligand treatment could have a synergistic effect [172]. Some PSMA antibodies are endocytosed and may release a linked therapeutic agent to the cytoplasm, which could allow the transport of larger molecules or simply reduce systemic effects. Alternatively, a PSMA-antibody-PARP conjugate could reduce the systemic adverse effects of conventional therapy and allow for higher concentrations in the target cells. Beneficial synergism may also occur when some other components of the DDR pathway are inhibited in combination with radioligand irrespectively of DDR mutation status of the tumor.

ADT upregulates PSMA expression [76]. The scheduling of PSMA homing therapies should be considered based on PSMA regulation and possibly androgen blockade lead-in doses could enhance the efficacy of PSMA targeting. A better understanding of inter- and intratumor heterogeneity of PSMA expression is important for the successful development of therapeutics targeting PSMA and developing optimal combinations. More detailed characterization of the regulators of PSMA expression and biological functions in prostate cancer is also of utmost importance for designing rational combination therapies. The first PSMA-based therapeutics are now likely to soon enter as a treatment option included in the standard clinical care of metastatic prostate cancer. The first phase III level evidence shows efficacy with ^177^Lu-PSMA-617 for heavily pretreated mCRPC patients [4], but the ongoing trials are evaluating PSMA-targeting theranostics for castration naïve metastatic prostate cancer compared to the current standard of care and may bring these agents into use as first-line therapy (Table 1). A deeper understanding of the determinants of sensitivity and mechanisms of resistance to the PSMA-based therapeutics is pivotal to improve the efficacy of these promising therapeutics. Recent advances in imaging and therapies put us a step closer to the chances of curing metastatic prostate cancer.

## Figures and Tables

**Table 1:** Registered therapeutic interventional clinical trials targeting PSMA in prostate cancer.

**Theranostics in Hormone Sensitive Prostate Cancer**						
**Radioactive Compound**	**Ab/Ligand**	**Regimen**	**Combination**	**Phase**	**Enrollment**	**NCT Number**
^177^Lu	PSMA-I&T	First Line		Feasibility Trial	5	NCT04297410
^64^Cu	DOTA-TLX592	First Line		Early Phase I	15	NCT04726033
^177^Lu	PSMA-617	First Line		Phase I & II	20	NCT04430192
^177^Lu	PSMA-I&T	First Line		Phase II	58	NCT04443062
^177^Lu	PSMA-617	First Line	Docetaxel	Phase II	140	NCT04343885
^177^Lu	PSMA-617	First Line	Antiandrogen	Phase III	1126	NCT04720157
**Theranostics in Castrate-Resistant Prostate Cancer**						
**Radioactive Compound**	**Ab/Ligand**	**Regimen**	**Combination**	**Phase**	**Enrollment**	**NCT Number**
^225^Ac	J591	Later		Early Phase I	18	NCT04576871
^225^Ac	Not Stated	Later		Early Phase I	20	NCT04225910
^227^Th	PSMA-TTC	Later		Phase I	157	NCT03724747
^177^Lu	PSMA-617	Later	Olaparib	Phase I	52	NCT03874884
^177^Lu	EB-PSMA-617	Later		Phase I	30	NCT03780075
^225^Ac	PSMA-617	Later		Phase I	30	NCT04597411
^177^Lu	PSMA-617	Later	Pembrolizumab	Phase I	43	NCT03805594
^177^Lu	CTT1403	Later		Phase I	40	NCT03822871
^225^Ac	J591	Later		Phase I	31	NCT03276572
^177^Lu	FC705	Later		Phase I	30	NCT04509557
^177^Lu	PSMA-617	Later		Phase I & II	10	NCT03828838
^177^Lu	PSMA-617	Later	Pembrolizumab	Phase I & II	37	NCT03658447
^177^Lu	PSMA-617	Later		Phase I & II	46	NCT03042468
^177^Lu	PSMA-R2	Later		Phase I & II	96	NCT03490838
^225^Ac	J591	Later		Phase I & II	105	NCT04506567
^177^Lu	PSMA-I&T	Post Doce or AA		Phase II	30	NCT04188587
^177^Lu	PSMA-617	Post Doce or AA		Phase II	210	NCT03454750
^177^Lu	PSMA-617	Post-Doce		Phase II	201	NCT03392428
^177^Lu	PSMA-617	Pre-Doce		Phase II	200	NCT04663997
^177^Lu	PSMA-617	Pre-Doce	Enzalutamide	Phase II	160	NCT04419402
^131^I	MIP-1095	Pre-Doce	Enzalutamide	Phase II	175	NCT03939689
^177^Lu	PSMA-617	Pre-Doce	BS/BSOC	Phase III	495	NCT04689828
^177^Lu	PNT2002	Pre-Doce		Phase III	415	NCT04647526
**Bispecific Antibodies in Castrate-Resistant Prostate Cancer**						
**Targets**	**Combination**		**Regimen**	**Phase**	**Enrollment**	**NCT Number**
PSMA & CD3			Later	Phase I	35	NCT02262910
PSMA & CD3			Later	Phase I	72	NCT04740034
PSMA & CD3	Pembrolizumab, Etanercept, Immunomodulating Agent or Monotherapy		Later	Phase I	288	NCT03792841
PSMA & CD3			Later	Phase I	86	NCT04104607
PSMA & CD3	Enzalutamide, Abiraterone or AMG 404 (PD1-inhibitor)		Pre-Doce	Phase I	105	NCT04631601
PSMA & CD28	Cemiplimab		Later	Phase 1 & 2	123	NCT03972657
**Chimeric Antigen Receptor (CAR) Cells in Castrate-Resistant Prostate Cancer**						
**CAR Cells**		**Regimen**	**Targeting**	**Phase**	**Enrollment**	**NCT Number**
PSMA-targeted CAR NK		Later	Prostate Cancer	Early Phase I	9	NCT03692663
PD-1-insensitive PSMA-targeted CAR T		Later	Prostate Cancer	Phase I	18	NCT04768608
TGFβ-insensitive PSMA-targeted CAR T		Later	Prostate Cancer	Phase I	50	NCT04227275
PSMA-targeted CAR T Co-expressing LIGHT		Later	Prostate Cancer	Phase I	12	NCT04053062
TGFβ-insensitive PSMA-targeted CAR T		Later	Prostate Cancer	Phase I	18	NCT03089203
PSMA-targeted CAR T		Later	Prostate Cancer	Phase I	13	NCT01140373
PSMA-targeted CAR T		Later	Prostate Cancer	Phase I	40	NCT04249947
PSMA-targeted CAR T		Later	PSMA-positive Solid Tumors	Phase I	35	NCT04633148
PSMA-targeted CAR T		Later	PSMA-positive Solid Tumors	Phase I & II	100	NCT04429451
**Antibody Drug Conjugates in Castrate-Resistant Prostate Cancer**						
**Molecule**			**Regimen**	**Phase**	**Enrollment**	**NCT Number**
PSMA-specific Antibody Linked to Monomethyl Auristatin E			Later	Phase I	10	NCT01414296
PSMA-specific Antibody Covalently Conjugated to Two Microtubule Disrupting Toxins			Later	Phase I	76	NCT04662580
PSMA-specific PSMA Antibody Linked to Monomethyl Auristatin E			Later	Phase II	9	NCT02020135
**Vaccine**						
**Description**			**Regimen**	**Phase**	**Enrollment**	**NCT Number**
RsPSMA Protein Vaccine with Alhydrogel Adjuvant			Later	Phase I	14	NCT00705835
TENDU Vaccine			First Line	Phase I	18	NCT04701021
Vaccine-based Immunotherapy Regimen (PrCa VBIR)			Later	Phase I	62	NCT02616185
PSMA Peptide-pulsed Autologous PBMC Vaccine Plus Interleukin-12			Later	Phase II	13	NCT00015977

AA = Antiandrogen, Ab = Antibody, BS/BSOC = Best supportive/Best standard of care, NCT Number = The National Clinical Trial number. Information was compiled by searching the ClinicalTrials.gov. The search was conducted under ‘Condition or disease’ of ‘Prostate cancer’ using ‘PSMA’ as ‘Other terms’ on 7 March 2021. All interventional trials using PSMA as a therapeutic target with reported statuses ‘Active, not recruiting’, ‘Not yet recruiting’, and ‘Recruiting’ are presented in the table.
